# Implementing mHealth Interventions in a Resource-Constrained Setting: Case Study From Uganda

**DOI:** 10.2196/19552

**Published:** 2020-07-13

**Authors:** Amanda J Meyer, Mari Armstrong-Hough, Diana Babirye, David Mark, Patricia Turimumahoro, Irene Ayakaka, Jessica E Haberer, Achilles Katamba, J Lucian Davis

**Affiliations:** 1 Department of Epidemiology of Microbial Diseases Yale School of Public Health New Haven, CT United States; 2 Uganda Tuberculosis Implementation Research Consortium Makerere University Kampala Uganda; 3 Departments of Social and Behavioral Sciences and Epidemiology School of Global Public Health New York University New York, NY United States; 4 Massachusetts General Hospital and Harvard Medical School Boston, MA United States; 5 Clinical Epidemiology and Biostatistics Unit Department of Medicine Makerere University College of Health Sciences Kampala Uganda; 6 Center for Methods in Implementation and Prevention Science Yale School of Public Health New Haven, CT United States; 7 Pulmonary, Critical Care and Sleep Medicine Section Yale School of Medicine New Haven, CT United States

**Keywords:** mHealth, implementation, tuberculosis, consolidated framework for implementation science, Uganda, framework, intervention, app

## Abstract

**Background:**

Mobile health (mHealth) interventions are becoming more common in low-income countries. Existing research often overlooks implementation challenges associated with the design and technology requirements of mHealth interventions.

**Objective:**

We aimed to characterize the challenges that we encountered in the implementation of a complex mHealth intervention in Uganda.

**Methods:**

We customized a commercial mobile survey app to facilitate a two-arm household-randomized, controlled trial of home-based tuberculosis (TB) contact investigation. We incorporated digital fingerprinting for patient identification in both study arms and automated SMS messages in the intervention arm only. A local research team systematically documented challenges to implementation in biweekly site visit reports, project management reports, and minutes from biweekly conference calls. We then classified these challenges using the Consolidated Framework for Implementation Research (CFIR).

**Results:**

We identified challenges in three principal CFIR domains: (1) intervention characteristics, (2) inner setting, and (3) characteristics of implementers. The adaptability of the app to the local setting was limited by software and hardware requirements. The complexity and logistics of implementing the intervention further hindered its adaptability. Study staff reported that community health workers (CHWs) were enthusiastic regarding the use of technology to enhance TB contact investigation during training and the initial phase of implementation. After experiencing technological failures, their trust in the technology declined along with their use of it. Finally, complex data structures impeded the development and execution of a data management plan that would allow for articulation of goals and provide timely feedback to study staff, CHWs, and participants.

**Conclusions:**

mHealth technologies have the potential to make delivery of public health interventions more direct and efficient, but we found that a lack of adaptability, excessive complexity, loss of trust among end users, and a lack of effective feedback systems can undermine implementation, especially in low-resource settings where digital services have not yet proliferated. Implementers should anticipate and strive to avoid these barriers by investing in and adapting to local human and material resources, prioritizing feedback from end users, and optimizing data management and quality assurance procedures.

**Trial Registration:**

Pan-African Clinical Trials Registration PACTR201509000877140; https://pactr.samrc.ac.za/TrialDisplay.aspx?TrialID=877

## Introduction

Mobile health (mHealth) and other electronic health (eHealth) interventions are becoming more common in low-income countries as advances in technology have enabled researchers and practitioners to engineer seemingly simple solutions to complex public health problems [1,2]. In many countries in sub-Saharan Africa, access to mobile phones is nearly universal, and mHealth apps such as mobile data collection software and SMS are showing great promise for enhancing capacity in resource-constrained health systems [3-5]. For example, mHealth technologies can promote treatment adherence [6-10], enhance access to and uptake of maternal and child health care services [11,12], and improve the quality and reliability of services by connecting external quality assurance teams directly to diagnostic instruments [13]. However, mHealth studies have met with mixed success in achieving their aims in these settings: many interventions have been piloted, yet few have made it into routine practice [1,3,14,15]. In many cases, it is unclear whether this lack of success reflects the ineffective implementation of these technologies or a lack of efficacy of the interventions themselves as most pilot studies do not report on implementation outcomes.

Implementation science is an emerging field that is highly relevant for answering such questions in mHealth by using interdisciplinary approaches, including quantitative and qualitative process evaluation. The goals of such investigations may be exploratory (ie, to plan for future implementation) or explanatory (ie, to identify causes of past implementation failures). Implementation science methods may be particularly useful for evaluating complex interventions, which are characterized by the presence of multiple interacting components that are a common feature of mHealth deployments. These methods are also well-suited to showing how changes in the implementation context may influence delivery [16]. Understanding the process and context can help improve fidelity to the core elements that drive the effectiveness of an intervention, as well as the adaptability of the intervention to the local setting [17].

**Figure 1:**
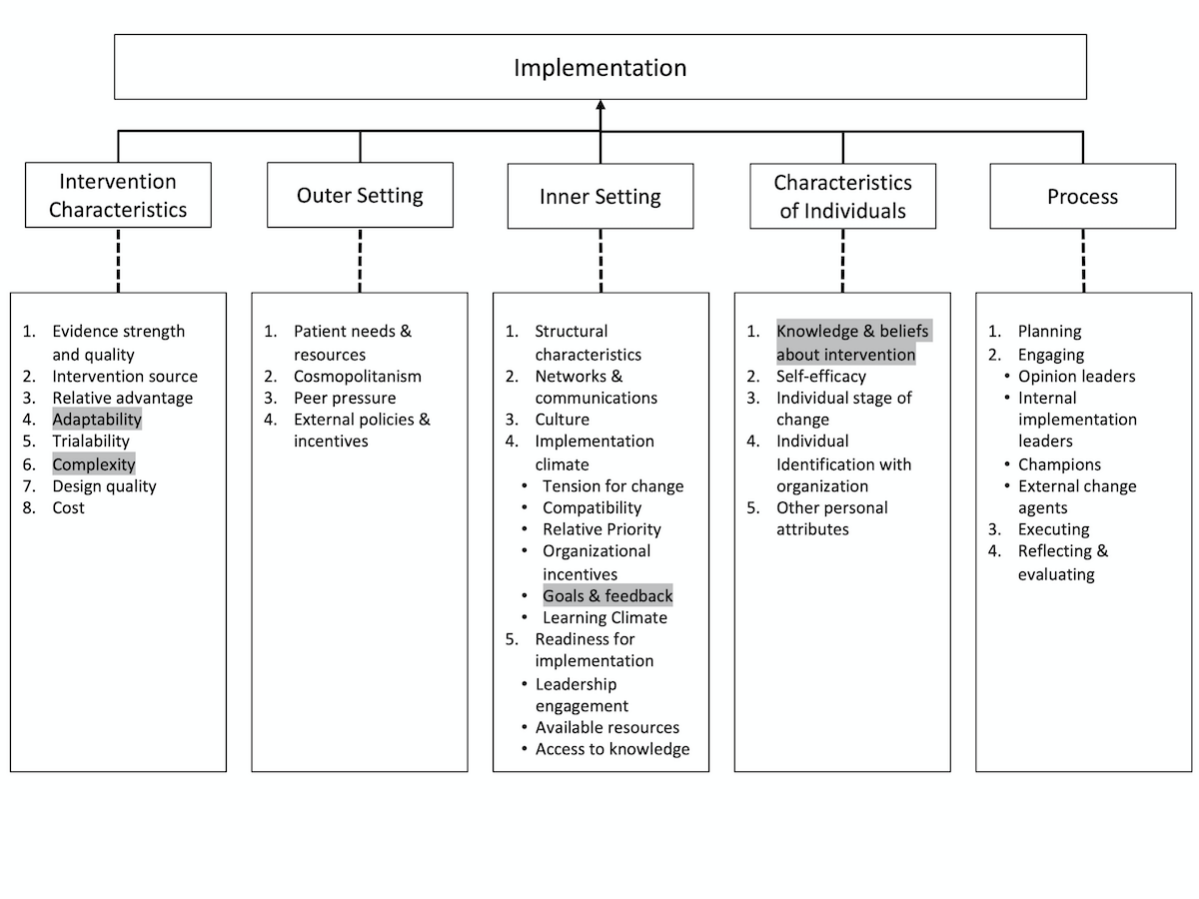
The Consolidated Framework for Implementation Research (CFIR), showing the five principal domains and highlighting 4 of the 39 underlying constructs/subconstructs that were identified as the most significant barriers and facilitators in the current analysis. Barriers and facilitators associated with constructs in the Outer Setting domain were not identified in this analysis. Barriers and facilitators associated with constructs/subconstructs in the Process domain have been previously described, and are not highlighted here [26,27].

The Consolidated Framework for Implementation Research (CFIR) is among the most widely used tools for planning and evaluating implementation in public health and population health [18]. It draws heavily on a rich literature on innovation in health and society [19,20]. According to the CFIR, factors at multiple levels may contribute to the success or failure of implementation, including (1) the characteristics of the intervention; (2) the inner setting where implementation is occurring; (3) the outer setting (ie, the economic, political, and social context within which an organization resides); (4) the characteristics of the implementers; and, finally, (5) the processes of implementation (Figure 1). Although the CFIR and other determinant frameworks have been widely applied to characterize implementation research [21-23], the CFIR has rarely if ever been used to examine the unique challenges associated with implementing mHealth interventions. Moreover, formal evaluations of mHealth implementation outcomes are especially important in low- and middle-income countries where information technology (IT) resources are more constrained, yet very few analyses of mHealth interventions in these settings have directly examined these challenges [24,25]. We used the CFIR to describe the process of implementing a complex mHealth intervention for tuberculosis (TB) case finding in Kampala, Uganda, in the context of a randomized controlled trial that showed no effect on the primary outcome. Our objectives in this study were to characterize the key challenges to introducing this intervention as planned to use a widely established implementation framework and to use the insights gained to propose generalizable, setting-appropriate solutions to mHealth implementation challenges.

## Methods

### Setting

Kampala, the capital city of Uganda, has approximately 3 million people. Rapid population growth in recent years has strained the capacity of the health care infrastructure to meet public health demands, and a high burden of infectious diseases compounds these problems. Given the high penetration of mobile phones and internet access, there has been interest in using new information and communication technologies (ICT) to support health infrastructure and improve patient care [4,28]. Although many mHealth and eHealth interventions have been piloted throughout Uganda, a shortage of trained ICT support personnel and a lack of public funding for digital health has limited adoption of technology into routine health practice [29].

### Developing the mHealth App

**Figure 2:**
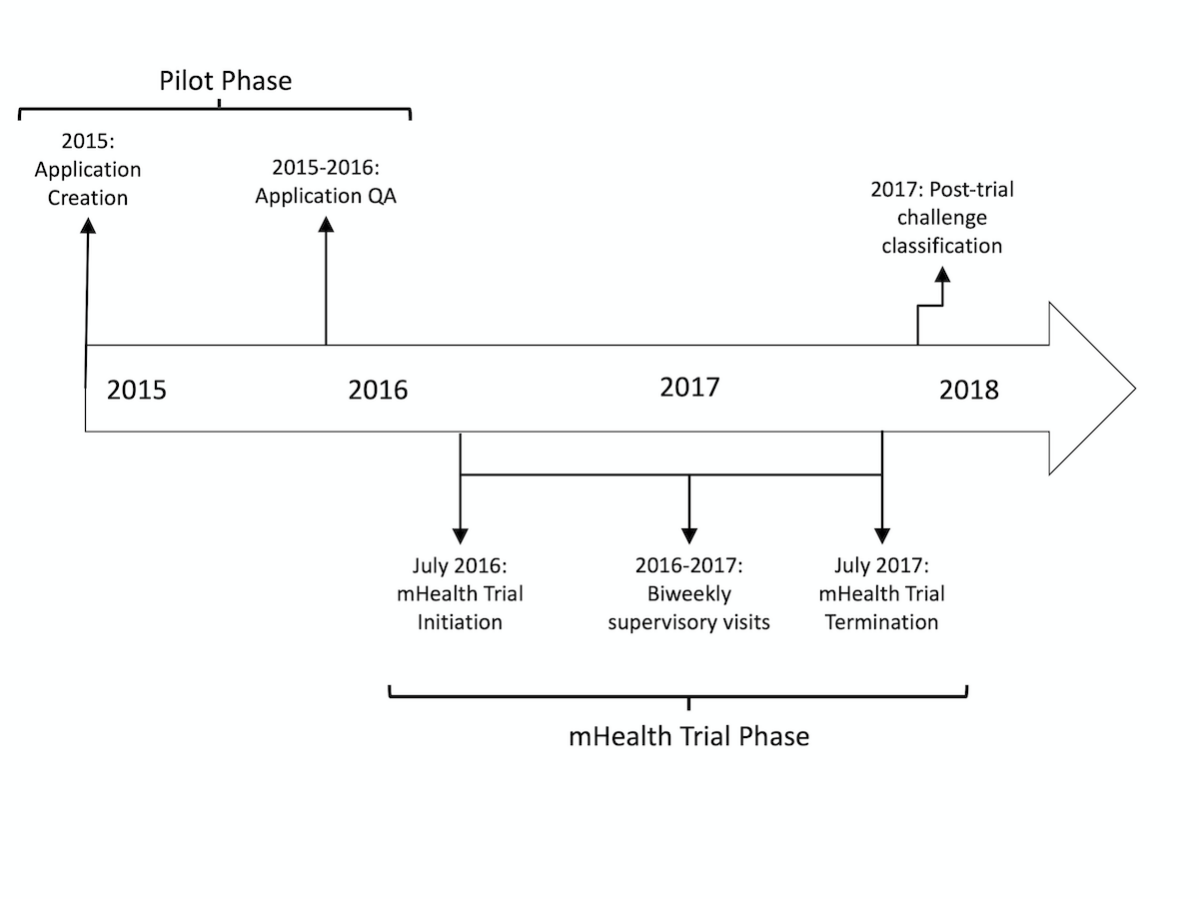
Timeline for the design and implementation of our mobile application. QA: quality assurance.

In 2015 (Figure 2), after a period of gathering mixed-methods data on user preferences and technology use [4], our study team, which included an experienced mHealth evaluator (JEH), an experienced mHealth implementer (DM), and commercial partners (Dimagi), created a customized mHealth app using CommCare software (Dimagi). The app was designed with two goals in mind: (1) to facilitate the implementation of household TB contact investigation using an evidence-based strategy for finding and treating close contacts with undiagnosed TB disease among household contacts of active TB patients; and (2) to evaluate this strategy in a household-randomized, controlled trial (called the “mHealth Trial,” Pan-African Clinical Trials Registration #201509000877140). The app incorporated several mHealth functions, including a survey app to collect participant information; digital fingerprinting (Biometrac) to help identify and track participants between their homes and multiple clinics (given widely recognized challenges with using a name, date of birth, and address for this purpose); and automated SMS messaging to communicate TB test results and follow-up instructions. We aimed to make the app easy for community health workers (CHWs) to use as they collected data during study-related household or clinic visits. For example, we added variable logic to display relevant questions based on participant characteristics and decision-support tools to guide on-site procedures and referrals. All data could be collected with or without internet connectivity; in-built functionality enabled subsequent, automated wireless syncing with a remote cloud-based server (CommCareHQ, Dimagi) via a third-generation (3G) mobile-broadband connection (Airtel Uganda) whenever connectivity was available. Local ICT staff (DB), customized the app in collaboration with an external ICT adviser (DM), a US-based data manager (AJM) and programmers and field officers from Dimagi.

As previously reported [30], the mHealth trial showed no improvement in the primary outcome of completing TB evaluation. Although household contacts and health workers spoke positively about the mobile app and text messaging, low rates of delivery and uptake of text-messaged instructions were observed [26,31]. Fingerprinting was also deemed acceptable, but implementation challenges resulted in inconsistent and declining use of the technology over time [27].

### Implementation of the Mobile Health App

We piloted the app for 10 months. During this time, we developed detailed quality assurance (QA) procedures by creating test cases that reflected all possible participant characteristics. Specifically, a team of staff members systematically verified all possible survey outcomes by entering all possible responses to all questions and verifying the observed behaviors on both health worker devices and in the back-end database. We repeatedly used these test cases to confirm expected app behaviors and to localize errors in variable logic or the functioning of hardware and software. We recorded all QA results in a spreadsheet, including errors encountered or barriers to implementing changes. During this time, we also received feedback from CHWs on user preferences or errors encountered. After rigorous testing and the adoption of changes requested by CHWs, we launched the app in July 2016 with the same staff and clinics for the start of the mHealth trial. Each time a modification was proposed to the app, such as an update to survey questions or variable logic, we conducted QA in a parallel app environment to verify that the changes operated as intended and to ensure that no other parts of the app were compromised. We released new versions of the app only after completing these QA procedures and confirming the full functionality of the revised app.

### Identifying Challenges to Implementation

In addition to the QA spreadsheet, study staff recorded implementation challenges in bi-weekly site visit reports, project management lists, and conference call minutes. A study coordinator (AJM) who oversaw programming of the survey app throughout the project reviewed these documents to identify key themes. We then mapped these themes using constructs and subconstructs from CFIR to classify key challenges to mHealth implementation. CFIR operationalizes these as 39 constructs/subconstructs within five previously described domains that together can be used to plan for and evaluate the success of implementation [21].

### Study Approvals

The mHealth trial protocol was approved by the School of Medicine Research Ethics Committee at Makerere University College of Health Sciences, the Uganda National Council for Science and Technology, and the Yale University Human Investigation Committee. In addition, the Uganda National TB and Leprosy Programme and the Health Directorate at the Kampala Capital City Authority approved project activities.

## Results

We reviewed implementation source documents collected between July 2015 and July 2017, including site visit reports, task lists recorded in an online project management tool, and minutes from weekly team conference calls, to identify barriers to implementation. We identified challenges relevant to three of the five principal CFIR domains: (1) *intervention characteristics*, (2) *inner setting*, and (3) *characteristics of individuals* who are implementing the intervention (Figure 1). We did not identify information on *outer setting* challenges related to technology. We largely excluded the implementation process domain from this analysis, as we have previously described these factors [26,27] concerning the key construct of execution (ie, the extent to which implementation went according to plan). We found that there was low fidelity for both SMS messages and fingerprinting but high levels of acceptability and feasibility, giving insight into the overall execution of the implementation strategy, therefore allowing us to exclude this domain from this analysis.

### Challenge: Adaptability and Complexity

Study staff identified limited adaptability of the mHealth app and the related hardware as a key challenge. According to CFIR, *adaptability* measures the degree to which an intervention can be altered to fit the local setting [18]. Although survey questions could be easily added or removed from the app, specialized software and hardware requirements for fingerprinting made the overall app less adaptable to the local setting. The need to have offline access to fingerprinting (eg, in the absence of internet connectivity) placed processing demands on the app that altered how participant data were collected. Offline access required custom re-coding of a sensitive section of the app that required the expertise of Dimagi engineers. The requirements for custom coding by external programmers reduced the app’s flexibility and adaptability and reduced its transparency to frontline ICT managers and its flexibility for design refinements based on input from end users. Additional challenges arose because digital fingerprinting required special tablets with specific hardware and software features that were not available in Uganda and had to be procured overseas. Replacing lost or broken tablets and peripheral connector cables, therefore, proved to be time-consuming and expensive, especially as cable connectors became obsolete as technology evolved towards using wireless connections for peripheral hardware. Increased rates of hardware failure over time ultimately reduced enrollment capacity for CHWs.

The complexity of the intervention and the logistics of its implementation also hindered the adaptability of the app. Within CFIR, *complexity* refers to implementation procedures that contain multiple interacting components, the perceived difficulty of implementing the intervention, and the number of steps required to implement an intervention [18]. Altering the app was complex because to ensure sustained functionality, every change had to be (1) assessed for both perceived usefulness to the overall study and risk to the functionality of the app, (2) tested using the QA procedures, and (3) introduced and implemented at the study sites. Because the last two steps were especially laborious, we managed all changes in batches, an approach that sometimes delayed updates by up to 4 months and limited our ability to iterate upon and adapt the app to local needs rapidly.

Similarly, creating appropriate logic for the automated SMS system proved surprisingly challenging and burdensome. Ultimately, over 50 new variables had to be created and continuously updated within the app as new clinical data became available during the evaluation process, all to make sure that the app correctly assigned participants to the appropriate end states required for them to receive timely and accurate messages. When we attempted to create test users to verify every possible pathway to every possible end state, this process added to the complexity of implementation, and the enormous number of possible pathways finally made this infeasible. The complex logic ultimately contributed to errors within the app and to mistimed SMS messages that were therefore never initiated, as previously described [26].

### Challenge: Knowledge and Beliefs About the Intervention

CHW knowledge and beliefs about the intervention also influenced its implementation. In CFIR, this construct focuses on users’ ideas about the intervention and the value that they place on the intervention itself [18]. Study staff reported that CHWs were enthusiastic about the technology to enhance TB contact investigation during training and initial implementation. However, after experiencing technology failures during the pilot phase, their trust in the app declined, and they reported developing an expectation that the technology, especially the fingerprinting features, would fail, even after the errors had been corrected. They began to avoid using what they perceived as problematic aspects of the technology whenever possible.

### Challenge: Goals and Feedback

Finally, the complex structure of the data coming out of the app slowed the development and implementation of a data management plan, making it difficult to provide timely feedback to study staff on progress towards key performance indicators for delivery of contact investigation, preventing the use of data for project management and quality assurance. *Goals and feedback*, a subconstruct in the inner setting domain, focuses on how well objectives are articulated and communicated to key stakeholders during implementation [18]. Software systems may not format data for easy management and timely dissemination to stakeholders, as others have observed [25]. In our project, consolidating, cleaning, analyzing, and disseminating data was time-consuming and required staff with advanced training in epidemiology and data management, as well as external data management and analysis apps, including Excel (Microsoft Corporation) and STATA (Stata Corporation). Extracting a master dataset required integration of 17 different worksheets using multiple unique identifiers in a complex hexadecimal format. Once created, reports could initially be run as needed, but when intermittent but automated software upgrades to CommCareHQ introduced new data export formats, the files had to be reprogrammed several times. These barriers limited the ability of local staff to interact with the data directly.

In addition to the challenges with data structure, missing data was another challenge that local staff identified as a barrier during implementation. The survey software did not differentiate between a failure to complete a service and a failure to simply record it, rendering many indicators inaccurate and requiring local study staff to spend substantial time working with CHWs to obtain missing data, reducing time to engage with them about overall implementation goals. For example, data were often missing when a participant had declined HIV testing, often because the app did not prompt CHWs to submit a form stating whether HIV testing was not provided or was not accepted by the participant. HIV results were found to be missing at other times when mobile tablets failed to synchronize with the cloud-based server because of a lack of connectivity with the 3G network, or depletion of a tablet’s pre-paid mobile service data plan. Missing data also interfered with dataset merges, preventing data updates until the missing data could be obtained, and preventing accurate projections or feedback on this key performance indicator.

### Discussion

Overall, mHealth interventions offer great potential to facilitate innovative solutions to complex public health problems. Nevertheless, the very technological advances that are designed to improve health also introduce challenges. Here, using a qualitative approach and drawing on the findings of prior process-oriented analyses [26,27], we identified three main challenges related to technology that other mHealth innovators are likely to encounter when creating and implementing technological solutions in resource-constrained settings: maintaining adaptability and reducing complexity; maintaining positive beliefs about the intervention among those who deliver it; and setting goals and providing feedback in a timely and comprehensible manner to key stakeholders.

Our first significant finding was that hardware and software requirements could severely limit the adaptability of mHealth technology in a resource-limited setting. Complex digital interventions, defined as those that perform multiple interacting functions, may require specialized hardware and software elements to function effectively. Hardware inevitably requires replacement, especially with intensive use, and software may become outdated as vulnerabilities emerge or operating systems are upgraded. Thus, sourcing the components and expertise locally to maintain mHealth systems is critical for these technologies to deliver on and sustain their promise. This is in tension with the fact that necessary technologies may be either less reliable or non-existent in local settings, even though these technologies have great potential to create meaningful change in local health systems [32]. A study in Malawi that evaluated an mHealth intervention for community case management of children with acute conditions identified similar challenges [33]. Although that program appeared efficacious, key stakeholders across the Malawian health sector identified a lack of integration with local programs and the limited capacity of local ICT and management as a significant barrier to the reliability and sustainability of the program.

Similarly, a recent systematic review of mHealth technologies in developing countries identified a lack of infrastructure and local equipment as a key barrier to scale-up of mHealth programs across multiple geographic locations [34]. As a result of our experiences, we would argue that if relevant local or regional design, manufacturing, and ICT support is routinely unavailable, this capacity should ideally be developed and nurtured to achieve the successful implementation of mHealth interventions. Utilizing experts from around the globe to develop ICT, computer engineering, and informatics programs is paramount to expand local human resources to support high-quality mHealth implementation on the ground in resource-constrained settings. This may help avert many of the challenges identified here before implementation even begins. However, it will likely take several years to build sufficient infrastructure in resource-constrained settings and utilizing external mHealth experts is not always feasible or acceptable. Therefore, we would recommend that architects of new mHealth programs simplify their designs, choose interchangeable hardware components and local suppliers, and train as many local ICT staff as possible as part of their program implementation. Furthermore, we recommend that mHealth designers apply the principles of CFIR throughout the design process to anticipate better and prevent challenges in the complexity and adaptability domains [21]. In practice, this could lead to a stepwise approach to implementation to ensure that each component is successfully introduced to ensure better sustainability.

Our second significant finding was that while end users and implementers may have an overall positive view of mobile health, trust in and use of specific platforms is likely to fade over time if end users experience more than occasional failures and frustrations. In our study, CHWs reported exploiting loopholes in the mobile app to avoid fingerprinting procedures because they found them to be highly error-prone [27]. Such failures introduced embarrassing delays for participants, further undermining the motivation of CHWs to use the technology. A previous study from South Africa similarly found that doctors and nurses were resistant to change from paper-based to electronic medical records because of a fear of disrupting patient care [35]. Our findings suggest that first impressions are critical and that rigorous quality assurance checks before “going live” with new technology may be more effective than pilot studies, as these can disappoint end users, sometimes permanently. This idea is captured in another theoretical model, the Technology Acceptance Model (TAM) [36]. The TAM states that an individual’s behavioral intention regarding the use of a technology is determined by the perceived usefulness and perceived ease of use, both of which may be impacted by end users’ first impressions of a technology and trust in that technology over time. After software updates or app changes, it is essential to rapidly conduct quality assurance testing and quickly resolve errors for the end user to ensure that the technology is useful and easy to use. Implementers must maintain the trust of end users by evaluating their beliefs and continually incorporating their input about the intervention at every step from design to implementation. In Sierra Leone, continuous feedback from end users to designers on how to eliminate errors and improve usability within the health care system was successfully used to refine a country-wide disease surveillance system in the wake of the Ebola crisis [37]. This is a promising approach for accelerating innovation and maintaining the satisfaction and trust of end users over time.

Our last significant finding was that the complexity of our data management system delayed feedback and prevented engaging stakeholders from achieving timelier, data-driven improvements to services. Future researchers and public health programs must consider ease of data management and accessibility to local managers when selecting software systems or considering customization. Once a software system has been selected, we recommend having a specific feedback plan in place in order to communicate goals to every level of stakeholder before implementation initiation to ensure that any mHealth technology selected will allow for appropriate communication between implementers and end users about implementation progress as well as ensure mHealth acceptability throughout. In addition, vendors that design and maintain survey software programs should prioritize consistency and usability in data management portals to facilitate continuous quality improvement activities by implementers.

This study had a few limitations. Although we collaborated with health administrators in the national TB program and local public health delivery network, we did not consult members of the Ministry of Health in charge of Uganda’s digital health strategy. This gap may explain why we did not identify elements from CFIR’s outer setting domain that may be highly relevant to implementing and scaling mHealth technologies. Although our focus in this project was on determining the effectiveness of our mHealth strategy before going to scale, researchers and public health officials within disease-specific areas should consider earlier consultation with policymakers to accelerate learning and adoption of best practices. Second, we used one particular software system and a limited range of hardware, which might limit the generalizability of this case study to other platforms. However, other projects in Sub-Saharan Africa have reported similar challenges with other mHealth platforms, leading us to believe that our findings transcend any specific software or hardware [24,25]. Third, new technologies are continually being developed and rapidly improved while academic evaluations such as this one move more slowly, potentially decreasing the relevance of our findings as newer technologies may not face the same challenges our study team experienced.

Our study also had important strengths. First, our study outlines operational and resource-related challenges to implementation that are specific to ICT in resource-constrained settings, complementing existing literature [38]. This work may also help address a critical gap in the TB literature, in that both the World Health Organization and a recent systematic review have called for structured evaluations of the implementation of digital health and TB [39,40]. Second, we used the CFIR to classify our challenges, thus increasing the generalizability of our results. The CFIR offers a standard set of principles for not only understanding but also anticipating implementation challenges independent of the setting. Third, we utilized information from a wide range of stakeholders, from implementing staff to end users over 4 years, resulting in a robust analysis of implementation challenges.

Overall, mHealth technologies have the potential to enhance public health programs regardless of disease or setting. However, the unique implementation challenges posed by these technologies should be examined, especially in low-resource settings, where ICT services have not yet proliferated. Implementation frameworks such as CFIR should be rigorously applied to evaluate mHealth interventions to measure not only their effectiveness but also key implementation outcomes such as fidelity and sustainability [15].

## Abbreviations

CFIR: Consolidated Framework for Implementation Research
CHW: community health workers
eHealth: electronic health
ICT: information and communication technologies
IT: information technology
mHealth: mobile health
QA: quality assurance
TB: tuberculosis
